# Introduction to LJPC Issue 9.5

**DOI:** 10.1080/17571472.2017.1364742

**Published:** 2017-09-06

**Authors:** Paul Thomas

Issue 9.5 of LJPC combines two essential aspects of modern-day primary care – good clinical care and leadership of integrated care.

Victoria Tzortziou Brown and seven co-authors review six innovations designed to bring specialist expertise about Chronic Kidney Disease and Chronic Obstructive Airway Disease right into primary care. They discuss transferable lessons for shared care at local, community level for other long-term conditions.

In the second of their series, the Hurst/Kelley-Patterson/Knapton team show how clever data gathering can help us to ask the right kind of questions about the design of integrated care. In this paper they reveal a close association between Emergency Department attendances and low patient satisfaction with their general practices. But there are some important twists to the story. Read it to get your mind thinking beyond the obvious.

Cheong, Alexiadou and Devendra elegantly review the causes and treatments of foot neuropathy. The discussion was triggered by a case study of a man who was found by the practice nurse to have absent monofilament sensation at his routine diabetic check. This is a helpful reminder for clinicians to be alert to silent things going wrong in the legs.

Basetti, Hodgson, Rawson and Majeed provide a helpful review of signs and symptoms of Scarlet Fever and how to distinguish it from other infections. Scarlet fever typically presents with high fevers, an erythematous sore throat, strawberry-like tongue and a sand-paper like rash that almost always originates from the groin and spreads bilaterally up the trunk to the axilla.

Carelli on Art. This month Francesco describes a Bologna exhibition of the work of Dali in which interactive and multimedia methods help visitors to experience his genius in different ways. He concludes that ‘these kinds of interactive and multimedia methods are already developing in teaching and learning modules in medicine …. And the Doctor as educator should feel as a protagonist in this new educative environment’. Amen to that.

